# Prevalence of Female Athlete Triad Risk Factors among Female International Volunteers and College Age-Matched Controls

**DOI:** 10.3390/ijerph19031223

**Published:** 2022-01-22

**Authors:** Annalisa N. Freire, Katie N. Brown, Stacie H. Fleischer, Dennis L. Eggett, Andrew R. Creer, Marlene I. Graf, Jenna Dyckman, Jennifer M. Turley, Susan Fullmer

**Affiliations:** 1 Department of Nutrition, Dietetics & Food Sciences, College of Life Sciences, Brigham Young University, Provo, UT 84602, USA; annalisafreire@outlook.com (A.N.F.); staciefleischer@gmail.com (S.H.F.); susan_fullmer@byu.edu (S.F.); 2 Department of Nutrition, Dietetics & Food Sciences, College of Agricultural and Applied Sciences, Utah State University, Logan, UT 84322, USA; marlene.graf@usu.edu; 3 Department of Statistics, College of Physical and Mathematical Sciences, Brigham Young University, Provo, UT 84602, USA; theegg@stat.byu.edu; 4 Department of Exercise Science & Outdoor Recreation, College of Science, Utah Valley University, Orem, UT 84058, USA; andrew.creer@uvu.edu; 5 Home and Community Department, Utah State University Extension, Logan, UT 84322, USA; jenna.dyckman@usu.edu; 6 Department of Exercise & Nutrition Sciences, Moyes College of Education, Weber State University, Ogden, UT 84408, USA; jturley2@weber.edu

**Keywords:** college students, female athlete triad, volunteers, bone mineral density, amenorrhea, food security, body satisfaction, weight loss

## Abstract

This study retrospectively compared the prevalence of factors related to the female athlete triad (low energy availability, secondary amenorrhea (SA), low bone mineral density (BMD)), and post-study BMD of female college students and female international volunteer missionaries (volunteers). Female college students (21–26 years) completed a survey that retrospectively assessed an 18-month study period (volunteer service or first 18 months of college); Diet History Questionnaire III (DHQ III) and Dual-Energy X-ray Absorptiometry (DXA) scan were optional. One-way ANOVAs and chi-squared distributions assessed group differences. Logistic regression assessed covariates of SA and BMD; corresponding odds ratios (OR) and confidence intervals (CI) were calculated. Statistical significance was set at *p* < 0.001. 3683 participants (58.8% volunteers, 31.5% non-volunteers, 9.8% others) provided complete survey data; 246 completed the DHQ III, and 640 had a post-study DXA scan. Volunteers had higher metabolic equivalent (MET) hours than non-volunteers and others (*p* < 0.001), and higher prevalence of food insecurity (*p* < 0.001) and SA (*p* < 0.001). Volunteers had higher odds of SA (OR = 2.17, CI = 1.75–2.62) than non-volunteers. Weight loss, body satisfaction, “other” weight loss methods, increased MET hours, and vomiting during the study period increased participants’ odds of SA. Participants’ average BMD Z-scores were within the expected range at all sites, with no significant group differences. Volunteers’ higher MET hours and higher prevalence of food insecurity and SA did not result in significantly lower post-study period BMD.

## 1. Introduction

The Female Athlete Triad (Triad) includes low energy availability (EA), menstrual irregularity (MI), and low bone mineral density (BMD) [1]. Low EA may result from dietary restriction, disordered eating, excessive physical activity, or may occur inadvertently [1]. Prolonged low EA can impair menstruation [2] and BMD [3]. BMD loss may not be fully reversible when optimal EA and menstruation are restored. In one study, female athletes who had previously experienced MI but had resumed normal menstruation for at least six years had lumbar spine BMD that was 15.2% lower than athletes with a history of eumenorrhea [4]. MI and low BMD independently increase risk for stress fractures and musculoskeletal injuries [5]. Such injuries incurred prior to menopause increase risk for post-menopausal fractures [4].

The Triad occurs in female high school, collegiate, and elite athletes [6,7]. In collegiate athletes, Triad components have the following prevalence estimates: Low EA (11.8–67%) [8,9], MI (16–36%) [8,10,11,12,13], and low BMD (Z-score < −1) (1–3.5%) [14].

The Triad may also be present in female international volunteer missionaries for the Church of Jesus Christ of Latter-day Saints (volunteers). Female volunteers are eligible to serve at age 19 and are assigned to serve for 18 months in locations worldwide. Volunteers follow a rigid schedule that includes arising early, engaging in religious study, then spending the day serving and teaching. Volunteers who expend significant energy walking and/or biking for transportation, and/or have decreased dietary intake due to limited invitations to eat in church or community members’ homes may experience low EA.

Though published research has not yet assessed MI among this population, a study of young women who served in the Peace Corps in Madagascar for two years reported that 27.7% had MI [15]. This is worth noting because young women serving in the Peace Corps may experience similar physical and emotional demands as volunteers. This study aims to retrospectively compare the prevalence of Triad components and risk factors of female volunteers and college students, and to compare post-study BMD among groups. We hypothesized that Triad components and risk factors would be present in female volunteers.

## 2. Materials and Methods

Females college students ages 21–26 years (*n* = 24,157) attending schools in Utah (Brigham Young University (BYU), Utah Valley University, Utah State University, Weber State University) and Idaho (Brigham Young University-Idaho, University of Idaho) were invited via email to participate in this study. At one institution, announcements were also made in classes and on social media. Invitations to participate included a study explanation, instructions, and risks and benefits of participating. Study consent was disclosed on the first page of the survey and was acknowledged by participants when they clicked to continue to survey questions. BYU’s Institutional Review Board reviewed and approved study procedures and all other universities signed authorization agreements.

Similar to previous retrospective research among college students [16], participants were asked to complete an online survey (Qualtrics, Provo, UT, USA) that assessed their health during a past time period, and their current health (time of survey completion). Participants were categorized as past female international volunteer missionaries for the Church of Jesus Christ of Latter-day Saints (volunteers), members of the Church of Jesus Christ of Latter-day Saints who did not serve a mission (non-volunteers), and female college students with other or no religious affiliation (other).

Volunteers answered questions specific to their 18-month volunteer service period. Volunteers aged 19 years and older often relocate away from home and live more independently. Non-volunteer and other participants answered questions about their first 18 months of college as it often requires a similar relocation, more independent living, and begins at a similar age.

Interested participants could choose to complete the optional online Diet History Questionnaire III (DHQ III) [17] and/or receive an optional Dual-Energy X-ray Absorptiometry (DXA) scan at BYU. Participants who provided written informed consent, agreed to provide a urine sample for a pregnancy test, and tested negative for pregnancy received a DXA scan.

The initial survey developed by the research team was reviewed by two independent researchers with survey-writing experience, piloted by 16 women who served as volunteers in the past, and six women who had not served as volunteers and refined based on their feedback. Changes in formatting and clarity were made. Instructions were also added to explain that volunteers should answer the questions based on what was most representative of their volunteer service period. Adding free-response questions allowed participants to explain their responses.

The survey assessed several Triad components or risk factors and used validated tools when available. Participants reported weekly hours of moderate (3–6 metabolic equivalents [METs]) [18], vigorous (6–10 METs), very vigorous (10–15 METs), and extremely vigorous (15–18 METs) physical activity. Responses were converted into MET hours per week (MET hours), a standard measure of physical activity [19]. Participants self-reported current or past clinically-diagnosed or self-diagnosed anorexia and/or bulimia [10]. The Six-Item Food Security Scale developed by the National Center for Health Statistics assessed food security [20]. Participants also reported study-period weight changes, as well as study period and post-study period weight satisfaction. Questions from the Low Energy Availability in Females Questionnaire (LEAF-Q) [21] assessed primary amenorrhea (menarche ≥ 15 years) and secondary amenorrhea ([SA], absence of menses for ≥3 consecutive months after menarche) [1]. The Perceived Stress Scale [22] classified participants’ study period stress levels. Participants also reported any study period stress fractures. Participants who were interested in receiving a DXA scan answered additional survey questions regarding osteoporosis risk factors.

The DHQ III, a validated and widely-used questionnaire developed by the National Cancer Institute [17] used participants’ reported frequency of consumption of foods to estimate usual study period daily macronutrient, micronutrient, and food group intake.

The Prodigy iDXA scanner (GE, Chicago, IL, USA) measured post-study period BMD and body composition. Z-scores for BMD (total body, lumbar vertebrae 1–4 (L1–L4), femoral neck, trochanter), and percent lean body mass (LBM) were recorded. In non-athletic populations, Z-scores < −2 are considered less than expected for age [23].

Statistical Analysis System (SAS version 9.4; SAS Institute Inc., Cary, NC, USA) was used for statistical analyses. Means and standard deviations were calculated to summarize continuous variables. Frequencies and percent of the total were calculated to summarize categorical data. Differences in group means were analyzed using one-way analysis of variance (ANOVA) with Tukey-Kramer post hoc tests. Chi-squared distributions analyzed group differences in categorical data. Logistic regression assessed covariates of menstrual health and BMD; corresponding odds ratios (OR) and confidence intervals (CI) were calculated. Due to the high number of statistical tests used, significance was set at *p* < 0.001 to minimize Type I errors.

## 3. Results

### 3.1. Participant Characteristics

Of the 24,157 eligible participants, 4015 females responded to the survey (16.6% response rate). Study respondents who initiated the survey but did not answer questions that identified group assignment (volunteer, non-volunteer, other) were eliminated (*n* = 332). Of the 3683 participants, 2164 (58.8%) were volunteers, 1159 (31.5%) were non-volunteers, and 360 (9.8%) were other. The majority (90.7%) of participants were White/Caucasian. Group characteristics are displayed in Table 1. There were no significant group differences in post-study height, weight, body mass index (BMI), or percent LBM. While 1367 participants (37.0%) volunteered to complete the DHQ III, 246 (6.7%) completed the questionnaire. Similarly, 1481 participants (40.2%) volunteered to have a DXA scan, and 640 (17.3%) completed a DXA.

### 3.2. Factors Related to Energy Availability

#### 3.2.1. Dietary Energy Intake

On average, volunteers consumed 2028 calories (kcals) per day during the study period. There were no significant group differences in intake of any nutrient or food group. When compared to non-volunteers and others, significantly more volunteers reported eating more during the study period than they did prior to the study period (Table 2).

#### 3.2.2. Eating Disorders

Group differences in clinically-diagnosed and self-diagnosed anorexia and bulimia were statistically significant, though likely were not practically significant (Table 2).

#### 3.2.3. Physical Activity

More volunteers reported exercising more during the study period than they did prior to the study period. They also engaged in more moderate-intensity physical activity than non-volunteers and others, and had higher average MET hours than non-volunteers (Table 3). Driving a car (45.9%) and walking (35.3%) were the most common modes of transportation for volunteers in this study. Group differences (*p* < 0.001) were likely due to lower rates of driving and higher rates of walking among non-volunteers, and more biking among volunteers (Table 3).

#### 3.2.4. Food Insecurity

Volunteer food insecurity prevalence (indicated by scores ranging from 2–6; 45%) was higher than non-volunteers (24%) and others (23%), *p* < 0.001. There were no significant group differences in post-study food insecurity (volunteers (37.4%), non-volunteers (37.2%), others (40.8%), *p* = 0.42).

#### 3.2.5. Weight Loss Methods, Weight Satisfaction, and Changes in Weight

Volunteers’ most common lifetime weight loss methods were very low-calorie diets (16.6%), fasting for non-religious purposes (16%), additional exercise beyond a regular training program (15.9%), and a high protein/low carbohydrate diet (15.8%). Over half (57.7%) of volunteers were dissatisfied with their body weight during the study period, but only 37% were dissatisfied after the study period (Table 4). Of note, fewer volunteers maintained their body weight during the study period than non-volunteers and others; 39.8% gained weight, 13.4% lost weight, and 35.3% gained and lost weight (Table 4).

### 3.3. Factors Related to Menstruation

#### 3.3.1. Participant Menstrual Regularity

Most (73.5%) volunteers reported achieving menarche at age 12–14 years. However, 14% achieved menarche at ≤11 years, 11.7% at ≥15 years (primary amenorrhea), and 0.1% had never menstruated. There were no group differences in age of menarche. Most volunteers (83.6%) indicated they could accurately recall menstruation frequency, which was similar to non-volunteers and others. Among volunteers, 43.2% reported menstruating less frequently during their volunteer service than prior to their volunteer service. More volunteers (30%) had SA compared to non-volunteers (16.5%) and others (25.7%), *p* < 0.001. After controlling for the lowest reported BMI, volunteers had 2.17 (95% CI = 1.75–2.62) greater odds of having SA than non-volunteers; others had 1.77 (95% CI = 1.3–2.40) greater odds of SA than non-volunteers. Volunteers were not at higher odds of SA than others (OR = 1.23, CI = 0.93–1.62).

#### 3.3.2. Actions Taken by Volunteers Who Experienced SA

Among volunteers who reported SA during the study period and described action(s) taken (*n* = 617), 66% took no action; another 15% were told that amenorrhea during their volunteer service was normal and to take no action; 15% spoke to a medical professional; 8% experienced SA prior to their volunteer due to medications or other causes; and 6% started taking oral contraceptives to regulate menstruation.

#### 3.3.3. Impact of Various Factors on Odds for SA

Participants who reported the following during the study period had increased odds of SA: losing weight (OR = 1.93, 95% CI = 1.04–3.58), neutral or satisfied with their body weight (OR = 1.48, 95% CI = 1.01–2.20), or lost weight via other methods not listed in the survey such as eliminating added sugars, eating small portions, or parasitic infection (OR = 1.65, 95% CI = 1.03–2.62). After removing outliers (<1% of data), odds of SA increased with increases in MET hours: increase of 10 MET hours (OR = 1.03, 95% CI = 1.02–1.05), increase of 25 MET hours (OR = 1.09, 95% CI = 1.06–1.12), increase of 50 MET hours (OR 1.18, 95% CI = 1.12–1.3), increase of 100 MET hours (OR = 1.40, 95% CI 1.13–1.54). Neither food insecurity (OR = 1.45, CI = 0.98–2.14), nor study period anorexia diagnosis (OR = 0.40, CI = 0.09–1.63) increased odds of SA. Non-volunteer and other participants who reported self-induced vomiting during the study period had increased odds of SA (OR = 2.78, 95% CI = 1.22–6.32). Volunteers who never attempted weight loss during the study period had reduced odds (OR = 0.66, 95% CI = 0.49–0.98) of SA compared to volunteers who attempted weight loss.

#### 3.3.4. Perceived Stress

The average participant perceived stress score was 19.2 ± 4.5 (moderate stress). More variation in stress levels was seen among volunteers (*p* < 0.0001); 15% reported low stress, 77.8% had moderate stress, and 6.7% had high stress. Almost all non-volunteers (97.6%) and others (97.4%) reported moderate stress.

### 3.4. Factors Related to Bone Mineral Density

Volunteers’ average L1-L4, femoral neck, trochanter, and total mean BMD Z-scores were within the expected range for age, and were not different from non-volunteers and others (Table 5). Among volunteers, 3.5% had BMD Z-scores below the expected range for age (Z-score ≤ −2), which was not significantly different from non-volunteers or others. Volunteers with SA did not have higher odds of BMD below the expected range for age.

Few volunteers (2.6%) reported a stress fracture during the study period and there were no significant group differences. Most volunteers (85.3%) reported no family history of osteoporosis; 11% reported previously having a stress fracture. There were no significant group differences in family history of osteoporosis or personal history of stress fractures. Few volunteers reported smoking for over one year (0.1%) or consuming >7 servings of alcohol per week for over one year (0.0%). Volunteers had lower rates of pregnancy (3.8%) than non-volunteers (6.8%) and others (17.8%), *p* < 0.0001.

Few volunteers (<1%) reported diabetes mellitus, cystic fibrosis, celiac disease, gastric bypass surgery, gastrointestinal surgery, Crohn’s disease, cancer, hyperparathyroidism, or rheumatoid arthritis. Though, 4.94% reported depression treated with medication and 1.44% reported polycystic ovarian syndrome. The most common medications used by volunteers were contraceptives (6.33%) and selective serotonin-reuptake inhibitors (3.45%). There were no group differences in disease incident or medication usage.

DHQ III data revealed that volunteers’ average study period intake of iron, magnesium, and vitamin D were below recommended dietary allowances [24,25,26]. Despite lower-than-recommended dairy intake, volunteers’ average calcium intake met the dietary reference intake [26]. There were no significant group differences in bone-related nutrient intake. Because few participants (*n* = 126) completed the DXA and the DHQ III, there was insufficient power to assess relationships between diet and BMD.

## 4. Discussion

This is the first study to investigate the prevalence of Triad components and risk factors in returned female international volunteer missionaries for the Church of Jesus Christ of Latter-day Saints. Despite increased odds of SA, higher physical activity, and higher prevalence of study period food insecurity, volunteers did not have a higher incidence of stress fractures or lower post-study period BMD. Lack of difference in BMD among groups may have also been influenced by high levels of physical activity among volunteers, which can promote increased BMD [27].

Though volunteers reported dietary energy intake that was within an expected range [28], a greater percentage of volunteers reported eating more than prior to the study period compared to non-volunteers and others. Volunteers eating in church or community members’ homes may have overeaten in attempts to not offend their host.

Volunteers reported clinically-diagnosed anorexia (0.5%) or bulimia (0.4%) during the study period was lower than reported among collegiate athletes (anorexia 1.7%, bulimia < 1% (13)), and college females (any eating disorder 5%) [29]. This was not unexpected because a history of eating disorders or eating disorder treatment must be reported in the pre-volunteer application. Applicants who reported no improvement or treatment may not have been recommended for volunteer service. A higher prevalence of self-diagnosed anorexia (9.6%) or bulimia (2.3%) may have been due to volunteers’ reluctance to disclose information to avoid the possibility of returning home to seek treatment.

Volunteers’ SA prevalence (30%) was similar to those of athletes (20.9–36%) [8,10,11,12,13] and females serving in the Peace Corps (27.7%) [15]. Several factors influenced participants’ odds of SA including weight loss, being neutral or satisfied with body weight, increases in MET hours, utilizing other weight loss methods not listed in the survey such as eliminating added sugar, eating small portions, or parasitic infection. The energy deficit required for weight loss may increase the risk for low EA and SA [1]. Most volunteers were dissatisfied with their weight during their volunteer service, likely influenced by weight gain (39.8% gained weight, 34.5% gained and lost weight). Among volunteers, increased odds of SA due to body satisfaction may have been influenced by corresponding weight loss and low EA. Weight loss methods such as eliminating added sugars and eating smaller portions likely contributed to lower energy intake and therefore low EA [1]. Parasitic infections may decrease nutrient absorption and increase vomiting and diarrhea resulting in low EA [30].

Increases in MET hours increased the odds of SA, which is consistent with previous evidence that suggests that increased exercise energy expenditure can contribute to low EA [1]. However, participants may have overestimated their physical activity [31]. Participants’ average MET hours were much higher than in previous studies (20–35 MET hours) [32,33] and recommendations to maintain general health (8.3–16.6 MET hours) [34].

Contrary to studies among athletes [4,35,36], volunteers’ increased odds of SA did not result in higher odds of BMD below the expected range for age. Though not assessed, volunteers’ duration of SA may have been too short or sporadic to impact BMD. Following volunteer service, some lost BMD may have been recovered. Volunteers’ study period (2.6%) and lifetime (11%) stress fractures rates were lower than reported among athletes (4.6–25%) [12,37,38]. Athletes may be at higher risk for stress fracture due to more intense physical activity and a longer duration of SA compared to volunteers.

Similar to research among college students [39], on average participants were moderately stressed. However, more volunteers reported having either low or high stress compared to non-volunteers and others. The prevalence of food insecurity among volunteers (45.6%) was higher than non-volunteers and others, and higher than or similar to previous studies of college students (31–46%) [40]. Food insecurity without corresponding increased odds of SA suggests that food insecurity duration may have been too short to impact EA and menstruation.

Large sample size, the inclusion of validated survey tools, objectively measuring BMD via DXA are strengths of this study. The self-reported nature of the study and the inability to provide DXA scans to all interested participants before the end of the semester or they lost interest are limitations. Though previous research validated retrospective dietary assessment [19], participants in this study may have been confused by conflicting instructions regarding the time period to report their dietary intake (email instructions from study personnel = 18-month time period; internal standardized DHQ III = past 12 months).

## 5. Conclusions

The data presented suggest that young adult women are at risk for the Triad; however, high physical activity, food insecurity, body dissatisfaction, and SA are especially pertinent to female international volunteers. Compared to non-volunteers, volunteers had greater odds of SA; however, there were no group differences in post-study period BMD.

Future prospective studies should investigate changes in BMD and other Triad components and risk factors during volunteer service. Participants’ odds of SA were impacted by weight loss, weight satisfaction, weight loss methods not listed in the survey such as eliminating added sugar, eating smaller portions, or parasitic infection, as well as increases in MET hours. Although volunteers did not have an increased risk for stress fractures or a higher prevalence of BMD below the expected range for age, SA was a common health problem that should be addressed. Training volunteers and volunteer service leaders about SA and related risk factors could help decrease SA incidence in volunteers. A high prevalence of Triad components and risk factors among all participants suggests that widespread education and resources for college-aged women may be warranted.

## Figures and Tables

**Table 1:** Post-study time period reported differences in sample population descriptive characteristics by group (*n* = 3683).

	All ^1^		Volunteer ^2^		Non-Volunteer ^3^		Others ^4^	
	*n*	Mean ± SD *n* (%)	*n*	Mean ± SD *n* (%)	*n*	Mean ± SD *n* (%)	*n*	Mean ± SD *n* (%)
Age	3534	23.3 ± 1.4	2081	23.6 ± 1.3	1105	22.8 ± 1.4 *	348	23.1 ± 1.6 *
Height (in)	3509	65.5 ± 2.8	2076	65.5 ± 2.8	1100	65.4 ± 2.8	333	65.5 ± 2.7
Height (m)		1.66 ± 0.07		1.66 ± 0.07		1.66 ± 0.07		1.66 ± 0.07
Weight (lb)	3506	146.6 ± 30.9	2069	147.1 ± 30.8	1103	144.7 ± 31.1	334	149.5 ± 31.4
Weight (kg)		66.6 ± 14.0		66.9 ± 14		65.8 ± 14.1		68 ± 14.3
BMI (kg/m^2^)	3500	24.0 ± 4.7	2067	24.1 ± 4.7	1100	23.7 ± 4.5	333	24.5 ± 4.8
%LBM	638	65 ± 6.6	305	64.8 ± 6.8	199	63.6 ± 6.4	13	62.5 ± 5.2
Ethnicity	3683		2164		1159		360	
White/Caucasian		3339 (90.7)		1987 (91.8)		1043 (90.0)		309 (85.8) *
Hispanic/Latina		242 (6.6)		132 (6.1)		77 (6.6)		33 (9.2)
Asian		132 (3.6)		69 (3.2)		41 (3.5)		22 (6.1)
African American		26 (0.7)		11 (0.5)		10 (0.9)		5 (1.4)
Pacific Islander		33 (0.9)		19 (0.9)		11 (1.0)		3 (0.8)
Native American/ Alaska Native		49 (1.3)		31 (1.4)		16 (1.4)		2 (0.6)
Other		8 (0.2)		3 (0.1)		3 (0.3)		2 (0.6)
University Attendance	3680		2163		1157		360	
BYU		2116 (57.5)		1429 (66.1)		677 (58.5)		10 (2.8)
BYU-Idaho		434 (11.8)		258 (11.9)		175 (15.1)		1 (0.3)
Utah Valley University		668 (18.2)		349 (16.1)		207 (17.9)		112 (31.1)
Utah State University		141 (3.8)		79 (3.7)		44 (3.8)		18 (5.0)
Weber State University		105 (2.9)		40 (1.9)		37 (3.2)		28 (7.8)
University of Idaho		206 (5.6)		4 (0.2)		12 (1.0)		190 (52.8)
Other		10 (0.3)		4 (0.2)		5 (0.4)		1 (0.3)

^1^ After answering the group assignment question, survey respondents became study participants and were not required to answer all remaining questions; therefore, the number of responses differed for each question. ^2^ Past female international volunteer missionaries for the Church of Jesus Christ of Latter-day Saints. ^3^ Members of the Church of Jesus Christ of Latter-day Saints who did not serve a mission. ^4^ Female college students with other or no religious affiliation. * Chi-Squared group distributions differed significantly (*p* < 0.0001).

**Table 2:** Differences in lifetime and 18-month study period reported medically-diagnosed and self-diagnosed anorexia and bulimia nervosa and self-reported changes to the amount of food consumed by group (chi squared) (*n* = 3683).

	All ^1^		Volunteer ^2^		Non-Volunteer ^3^		Others ^4^		*p*-Value
	*n*	Mean ± SD *n* (%)	*n*	Mean ± SD *n* (%)	*n*	Mean ± SD *n* (%)	*n*	Mean ± SD *n* (%)	
Lifetime									
Clinically-Diagnosed Anorexia	3682	118 (3.2)	2163	45 (2.1)	1159	56 (4.8)	360	17 (4.7)	<0.0001
Clinically-Diagnosed Bulimia	3678	68 (1.9)	2160	25 (1.2)	1158	30 (2.6)	360	13 (3.6)	0.0005
Study Period									
Clinically-Diagnosed Anorexia	3484	38 (1.1)	2135	11 (0.5)	1046	18 (1.7)	303	9 (3.0)	<0.0001
Self-Diagnosed Anorexia	3884	212 (6.1)	2135	102 (4.8)	1046	81 (7.7)	303	29 (9.6)	0.0001
Clinically- Diagnosed Bulimia	3484	27 (0.8)	2134	9 (0.4)	1047	12 (1.2)	303	6 (2.0)	0.004
Self-Diagnosed Bulimia	3484	98 (2.8)	2134	50 (2.3)	1048	35 (3.3)	302	13 (4.3)	0.0726
Changes in Food Consumption	3313		2059		982		272		<0.0001
Ate less		800 (24.2)		331 (16.1)		377 (38.3)		92 (33.8)	
Ate more		1581 (47.7)		1256 (61.0)		247 (25.2)		78 (28.7)	
No Changes		862 (26.0)		422 (20.5)		343 (34.9)		97 (35.7)	
Don’t know		70 (2.1)		50 (2.4)		15 (1.5)		5 (1.8)	

^1^ After answering the group assignment question, survey respondents became study participants and were not required to answer all remaining questions; therefore, the number of responses differed for each question. ^2^ Past female international volunteer missionaries for the Church of Jesus Christ of Latter-day Saints.^3^ Members of the Church of Jesus Christ of Latter-day Saints who did not serve a mission. ^4^ Female college students with other or no religious affiliation.

**Table 3:** Differences in weekly MET hours of physical activity, hours in physical activity categories (ANOVA), and changes in physical activity (chi-squared) by group during the 18-month study period (*n* = 3683).

	All ^1^		Volunteer ^2^		Non-Volunteer ^3^		Others ^4^	
	*n*	Mean ± SD *n* (%)	*n*	Mean ± SD *n* (%)	n	Mean ± SD *n* (%)	*n*	Mean ± SD *n* (%)
Total MET Hours/Week	3256	93.8 ± 80.9	1972	108.8 ± 81.4	1003	65.2 ± 65.0 *	281	90.6 ± 98.4 ***
Physical Activity by Category (hours/week)								
Moderate Intensity	3328	11.3 ± 9.3	2010	14.3 ± 9.7	1025	6.5 ± 5.6 *	293	7.7 ± 7.5 *
Vigorous Intensity	3402	3.4 ± 4.1	2090	3.7 ± 4.2	1018	2.8 ± 3.5 *	294	3.8 ± 5.0 **
Very Vigorous Intensity	3383	1.0 ± 2.1	2081	1.0 ± 2.14	1012	0.8 ± 1.7	290	1.4 ± 2.9 **
Extremely Vigorous Intensity	3379	0.3 ± 1.1	2077	0.2 ± 1.01	1014	0.3 ± 0.9	288	0.5 ± 1.7 ***
Physical Activity Changes ****	3311	*n* (%)	2058	*n* (%)	981	*n* (%)	272	
Exercised more		1262 (38.1)		857 (41.6)		311 (31.7)		94 (34.6)
Exercised less		1410 (42.6)		863 (41.9)		432 (44.0)		115 (42.3)
No Change		596 (18.0)		306 (14.9)		228 (23.2)		62 (22.8)
Don’t know		43 (1.3)		32 (1.6)		10 (1.0)		1 (0.4)
Most Common Modes of Transportation ****	3611		2156		1122		333	
Driving a Car		1461 (40.5)		989 (45.9)		310 (27.6)		162 (48.7)
Walking		1609 (44.6)		760 (35.3)		716 (63.8)		133 (40.0)
Public Transportation		281 (7.8)		193 (8.95)		60 (5.4)		28 (8.4)
Biking		236 (6.5)		206 (9.6)		26 (3.3)		4 (1.2)
Other		24 (0.7)		8 (0.37)		10 (0.9)		6 (1.8)

^1^ After answering the group assignment question, survey respondents became study participants and were not required to answer all remaining questions; therefore, the number of responses differed for each question. ^2^ Past female international volunteer missionaries for the Church of Jesus Christ of Latter-day Saints. ^3^ Members of the Church of Jesus Christ of Latter-day Saints who did not serve a mission. ^4^ Female college students with other or no religious affiliation. * Significantly different than the Volunteer group (*p* < 0.001). ** Significantly different than the Non-volunteer group (*p* < 0.001). *** Significantly different than the Volunteer and Non-volunteer groups (*p* < 0.0001). **** Chi-Squared group distributions differed significantly (*p* < 0.0001).

**Table 4:** Differences in weight satisfaction and weight change by group during the 18-month study and post-study time periods (chi squared) (*N* = 3683).

	All ^1^		Volunteer ^2^		Non-Volunteer ^3^		Others ^4^	
	*n*	Mean ± SD *n* (%)	*n*	Mean ± SD *n* (%)	*n*	Mean ± SD *n* (%)	*n*	Mean ± SD *n* (%)
Total MET Hours/Week	3256	93.8 ± 80.9	1972	108.8 ± 81.4	1003	65.2 ± 65.0 *	281	90.6 ± 98.4 ***
Physical Activity by Category (hours/week)								
Moderate Intensity	3328	11.3 ± 9.3	2010	14.3 ± 9.7	1,025	6.5 ± 5.6 *	293	7.7 ± 7.5 *
Vigorous Intensity	3402	3.4 ± 4.1	2090	3.7 ± 4.2	1,018	2.8 ± 3.5 *	294	3.8 ± 5.0 **
Very Vigorous Intensity	3383	1.0 ± 2.1	2081	1.0 ± 2.14	1,012	0.8 ± 1.7	290	1.4 ± 2.9 **
Extremely Vigorous Intensity	3379	0.3 ± 1.1	2077	0.2 ± 1.01	1014	0.3 ± 0.9	288	0.5 ± 1.7 ***
Physical Activity Changes ****	3311	*n* (%)	2058	*n* (%)	981	*n* (%)	272	
Exercised more		1262 (38.1)		857 (41.6)		311 (31.7)		94 (34.6)
Exercised less		1410 (42.6)		863 (41.9)		432 (44.0)		115 (42.3)
No Change		596 (18.0)		306 (14.9)		228 (23.2)		62 (22.8)
Don’t know		43 (1.3)		32 (1.6)		10 (1.0)		1 (0.4)
Most Common Modes of Transportation ****	3611		2156		1122		333	
Driving a Car		1461 (40.5)		989 (45.9)		310 (27.6)		162 (48.7)
Walking		1609 (44.6)		760 (35.3)		716 (63.8)		133 (40.0)
Public Transportation		281 (7.8)		193 (8.95)		60 (5.4)		28 (8.4)
Biking		236 (6.5)		206 (9.6)		26 (3.3)		4 (1.2)
Other		24 (0.7)		8 (0.37)		10 (0.9)		6 (1.8)

^1^ After answering the group assignment question, survey respondents became study participants and were not required to answer all remaining questions; therefore, the number of responses differed for each question. ^2^ Past female international volunteer missionaries for the Church of Jesus Christ of Latter-day Saints.^3^ Members of the Church of Jesus Christ of Latter-day Saints who did not serve a mission. ^4^ Female college students with other or no religious affiliation. * Significantly different than the Volunteer group (*p* < 0.001). ** Significantly different than the Non-volunteer group (*p* < 0.001). *** Significantly different than the Volunteer and Non-volunteer groups (*p* < 0.0001). **** Chi-Squared group distributions differed significantly (*p* < 0.0001).

**Table 5:** Group Differences in Post-Study Period Bone Mineral Density and Z-Scores Measured by DXA (ANOVA) (*n* = 640).

	All ^1^		Volunteer ^2^		Non-Volunteer ^3^		Others ^4^	
	*n*	Mean ± SD *n* (%)	*n*	Mean ± SD *n* (%)	*n*	Mean ± SD *n* (%)	*n*	Mean ± SD *n* (%)
Spine								
L1-L4 BMD (g/cm^2^)	638	1.22 ± 0.13	422	1.23 ± 0.12	203	1.23 ± 0.13	13	1.16 ± 0.12
L1-L4 Z-Score	638	0.29 ± 0.96	422	0.30 ± 0.94	203	0.30 ± 0.94	13	−0.18 ± 0.78
Femur								
Neck BMD (g/cm^2^)	640	1.07 ± 0.14	424	1.07 ± 0.12	203	1.07 ± 0.14	13	1.09 ± 0.13
Neck Mean Z-Score ^4^	632	0.16 ± 0.96	421	0.21 ± 0.94	198	0.09 ± 0.98	13	−0.34 ± 0.91
Troch BMD (g/cm^2^)	640	0.83 ± 0.12	424	0.83 ± 0.13	203	0.83 ± 0.12	13	0.79 ± 0.12
Troch Mean Z-Score	632	−0.26 ± 1.02	421	−0.23 ± 1.03	198	−0.13 ± 1.01	13	−0.58 ± 0.97
Total Mean BMD	633	1.07 ± 0.13	420	1.07 ± 0.12	100	1.02 ± 0.13	13	1.02 ± 0.13
Total Mean Z-Score	632	0.43 ± 0.99	421	0.47 ± 0.99	198	0.35 ± 1.00	13	0.89 ± 1.01
Total Body								
Total BMD score	640	1.22 ± 0.10	424	1.23 ± 0.10	203	1.23 ± 0.09	13	1.18 ± 0.11

^1^ Past female international volunteer missionaries for the Church of Jesus Christ of Latter-day Saints. ^2^ Members of the Church of Jesus Christ of Latter-day Saints who did not serve a mission. ^3^ Female college students with other or no religious affiliation. ^4^ Average of both the right and left sides. No statistical differences between groups.

## Data Availability

The data presented in this study are available on request from the corresponding author.
